# Real-time field sports scene classification using colour and frequency space decompositions

**DOI:** 10.1007/s11554-014-0437-7

**Published:** 2014-06-28

**Authors:** Rafal Kapela, Kevin McGuinness, Noel E. O’Connor

**Affiliations:** 1 Department of Computer Engineering, Poznan University of Technology, Poznan, Poland; 2 CLARITY, Dublin City University, Dublin 9, Ireland

**Keywords:** Real-time scene recognition, Fourier transform, Field sports

## Abstract

This paper presents a novel approach to recognize a scene presented in an image with specific application to scene classification in field sports video. We propose different variants of the algorithm ranging from bags of visual words to the simplified real-time implementation, that takes only the most important areas of similar colour into account. All the variants feature similar accuracy which is comparable to very well-known image indexing techniques like SIFT or HoGs. For the comparison purposes, we also developed a specific database which is now available online. The algorithm is suitable in scene recognition task thanks to changes in speed and robustness to the image resolution, thus, making it a good candidate in real-time video indexing systems. The procedure features high simplicity thanks to the fact that it is based on the very well-known Fourier transform.

## Introduction

Despite many years of active research and considerable progress, the problem of automatically identifying scene classes in sports video both efficiently and accurately remains largely unsolved. Many existing techniques rely on complex and computationally demanding visual features, which makes them expensive to use on large volumes of archived content, and infeasible for real-time analysis. Most existing techniques are, in addition, focused entirely on a single sports genre, primarily soccer, and do not generalize easily to alternative genres. This paper examines the tradeoffs between efficiency and accuracy in sports scene classification algorithms, and proposes a simple efficient algorithm that provides state-of-the-art performance, yet remains sufficiently generic to be used on a wide range of sports genres.

Efficient algorithms for sports scene classification have many potential applications. Automatic sports scene classifiers can produce annotations that can be used to index large volumes of archived content. These annotations can assist in both browsing of such content, by helping direct users to interesting portions of content, and in search, by allowing users to filter or boost certain specific scene types. Scene classification is also useful for automatic sports summarization. General scene type information from such classifiers may also be used as prior knowledge for genre- specific event classification. Algorithms with modest computational requirements are not only more cost effective for indexing large volumes of archived content, but are also essential if they are to be used on modern mobile devices or used to perform real-time processing on sports video as it is generated [8].

Given the potential commercial applications, scene classification sports video has been widely studied and many approaches have been proposed. Several authors have proposed general approaches for scene classification that apply to a wider variety of sports-related video. Pei and Chen [25] used low-level features such as field colour distribution, field colour percentage, colour histogram similarity or colour-based object location verification to detect scenes and events in tennis and baseball games. For very small dataset (half an hour video), they achieved 60–96 % recall ratio in recognition scenes such as serves, close-ups, in/out-field shots, etc. Zhong and Chang [39] using colour-based filtering and then colour and edge certification techniques attempted to analyse index tennis and baseball sport videos in real time. For one hour only video dataset, they achieved about 80 % scene recognition accuracy. Tong et al. [33] focus on classification of replay vs. non-replay shots, and of medium, close-up, and out-of-field shots. Their system uses a decision tree-based classifier and simple low-level general features, such as global gray-level co-occurrence, and mid-level sports-specific visual features such as field ratio as a proportion of dominant colour, head area using a skin colour detector, and object scale determined by segmentation of the field area. Mei et al. [22] focus on play vs. non-play and key event detection via mosaic reconstruction, again using a combination of simple global visual features (colour histograms, camera motion) and sports-specific mid-level features. Barnard and Odobez [3] consider camera-type events (medium shot, close up, long shot, etc.) and play-type events (play, nonplay, and replay) using layered HMMs to model the statistical dependencies between mid-level and high-level events. The authors again use simple visual features based on colour and texture. They evaluate their system using only rugby content and achieve detection accuracies of 67–79 Bayesian belief network method for automatically finding and indexing exciting sequences in sports video, and genre-specific events using simple global visual features such as dominant green colour pixel ratio, hue histograms, and skin colour detectors. Choroś and Pawlaczyk [6] using features such as pace of audio narration, camera motion, light changes, action dynamism and existence of special effects (logo transitions) proposed content-based scene detection system for TV news. Their system scores 51–100 % of recall and 62–82 % of precision factors.

There was also vast investigation on scene analysis in real time. Most trends however tend to focus on motion estimation and motion-based object tracking for further parametrization of the video broadcast. In [37], motion object extraction based on optical flow analysis is presented. Among other real-time optical flow-based object extraction, this one seems to be applicable in scenarios where the system has to deal with analysis in compressed domain in real time. There are also available fast algorithms for motion detection for decompressed domain like [19] or [20] that can work on embedded platforms, so having other than MPEG2 compression does not constitute a problem in this case. System architect however has to be aware that in the latter solutions there is a need to have real-time video decompression and enhancement module which is usually a hardware module (especially in embedded systems) [30] or [31]. The output of these algorithms can be used in player tracking application based on optical flow like the one presented in [38]. Work presented there seems to be applicable for variety of sports and even surveillance videos but is not claimed to be working in real time. Another, smaller trend that can be observed in real-time video analysis applications is object detection based on various approaches. In [14], authors focus on existing object detection algorithms to make them applicable for real-time applications using Compute Unified Device Architecture (CUDA). The article in particular focuses on detection by statistical classifiers based on boosting. The implemented procedure is proven to work much faster than CPU equivalents of the same algorithms. CUDA is a very good tool when it comes to real-time video or image analysis. In [18], authors use GPU computing to detect and segment from the background players of a football game. This is done based on colour segmentation so that assumption has to be made that the pitch colour falls into a particular range of hue colour coefficient. The application deals with the problem in real time and seems to be applicable in the applications where another example of object or scene categorization in real time is presented in [1] where SIFT features are taken for real-time matching, thus providing a fast procedure to compare or detect objects in the image once all the features are calculated. Finally, there are investigations like [36] for real-time analysis of the robo-sports played in artificial arena. This trend seems to be based on the analysis of real team matches where different heuristics are extracted to provide the base for further investigation on the efficient control of swarm of agents in a dynamically changing environment.

There are several limitations of the state-of-the-art approaches. Clearly, the genre-specific approach is limited in its applicability, requiring the development of new bespoke algorithms for each new sport to be supported. Several algorithms only make use of very simple low-level features such as global histograms and global motion; recent developments in general scene classification suggest that modern local features such as the scale invariant feature transform (SIFT) [21], speeded up robust features (SURF) [4], and histograms of oriented gradients (HoG) [10], combined with aggregation techniques such as bag of visual words, vectors of locally aggregated descriptors (VLAD) [15], or Fisher kernels [26], can often dramatically improve performance over such simple features, as evidenced by their widespread adoption in classification challenges like PASCAL VOC [12]. Many algorithms also make use of sports-specific mid-level features such as grass-area ratio, which lack theoretical justification and can often be brittle, or rely on external sources of textual information to achieve reasonable accuracy rates. Finally, many of the above described approaches are computationally demanding, and there is very little discussion in the literature about the typical computational demands of such systems or how they should be optimized for practical use. Quite commonly CUDA technology is used as a solution to these problems but it involves usage of specialized equipment, that can be expensive.

**Fig. 1:**
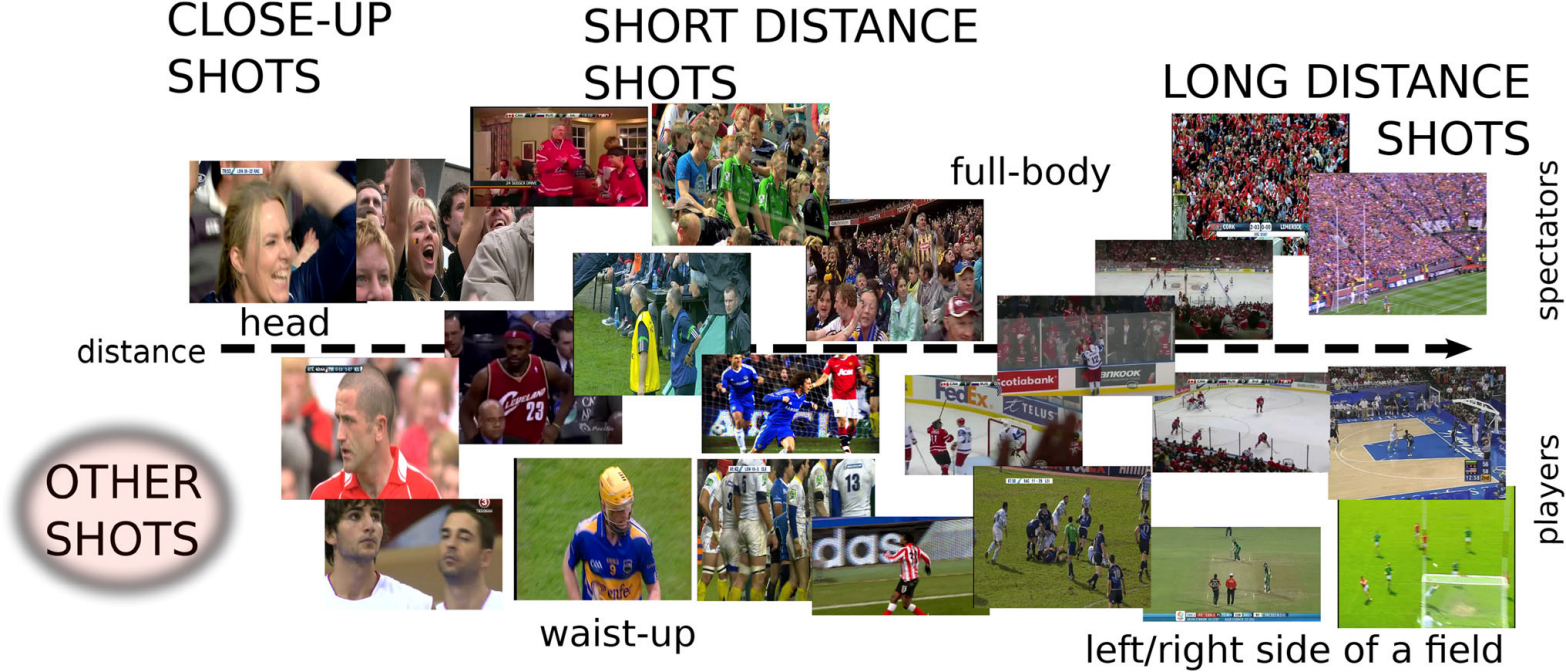
Types of the most common video shots present in broadcasts nowadays; note that some of the images cannot be interchangeably assigned to players/spectators class

This paper proposes a very general feature extraction algorithm that can potentially be used for scene classification in a wide variety of sports videos. Our method is based on radial basis decompositions of a colour address space followed by spatially localized texture decompositions using Gabor wavelets in frequency space. These decompositions are aggregated in various ways to produce frame-level descriptors that are then used to train a support vector classifier. Unlike previous work in sports scene classification, we pay particular attention to computational requirements, deriving an algorithm that is comparable with the state-of-the-art in terms of accuracy, but which requires significantly less computational resources. For concreteness, our experimental evaluation focuses on field sports video as this covers multiple individual sports such as basketball, soccer, rugby, football, all genres of hockey, etc.; however, as our descriptor avoids using sports video-specific mid-level features, like grass ratios, it is sufficiently general to be applicable in other contexts.

The remainder of the paper is organized as follows. Section 2 describes the scene classification scheme that we use for fields sports. Section 3 discusses in detail the proposed feature extraction and classification algorithm, and describes several variants thereof. Section 4 presents the results of our experiments and compares the proposed algorithm variants with other state-of-the-art scene classification descriptors. Section 5 concludes the paper and discusses potential future work.

## Field sport scene types

For a given sport scene detection task, it is unrealistic to consider that the same indexing system will work with the same accuracy for all sport genres. However, we believe that there do not exist any reasonable barriers that can prevent the use of scene description algorithm presented herein in other kinds of sports or even in more general tasks like scene detection or recognition. For presentation purposes, the work proposed herein aims to set some meaningful boundary on a generic approach to field sports video event detection. At the same time, we would like to limit our scope to some extent so as not to be very content specific. The chosen domain is field sport broadcasts, encompassing all sports genres that fall within this ambit. Since most field sports have similar characteristics, broadcasters use the same scene compilation techniques to present the action in the field to the viewer. Thus, once a robust and accurate algorithm is developed, it can be applied to different kinds of field sports without any modification.

**Fig. 2:**
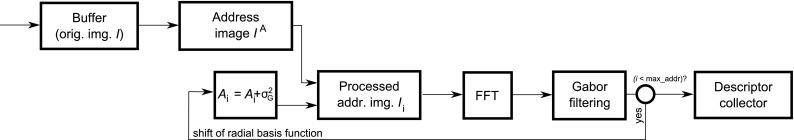
The block diagram of the algorithm

Figure 1 presents the most common video shot types. As can be seen, we sort all the shots from all types of field video sports based on the perceived distance between the camera and the object presented in the shot. Another characteristic on which these shots can be categorized is the fact, that they can present either player(s) or spectator(s). In addition, we introduce a third category which includes all the shots not related to the previous two classes.

Note, that the separation between players and spectators is much more vague than the separation based on the perceived distance, since quite often the shot can present both categories at the same time. The other problem is that the number of players presented can be high (e.g., defence under the basket in basketball or a scrum in rugby) so it could be hard for the algorithm to classify these situations as accurately as for the shots that present for example a close-up of a single player on the simple background. A similar situation can occur in the head shots type, where face recognition algorithms are needed to distinguish if the presented person is a player or spectator. The categorization with respect to the perceived distance to the object is much more reliable. But here the algorithm has to have some flexibility since for example, because given the size of the pitch/ice rink and the locations of the cameras it could be hard to recognize shots that present left/right sides of the play area.

For our experiments, we have chosen fourteen different classes that represent most of the situations presented on the field during a match. To evaluate the generalization properties of the algorithm, some of the classes were split into three subclasses: shot with simple background (a dominant colour easily distinguishable), shot with complex background (no dominant colour in the background) and a mixture of the two:

- close up shot head with simple background (CL_H_S)
- close up shot head complex background (CL_H_C)
- close up shot head mixture background (CL_H)
- close up shot waist up simple background (CL_WU_S)
- close up shot waist up complex background (CL_WU_C)
- close up shot waist up mixture background (CL_WU)
- short distance shot presenting player(s) simple background (S_P_S)
- short distance shot presenting player(s) complex background (S_P_C)
- short distance shot presenting player(s) mixture background (S_P)
- short distance shot presenting spectators (S_S)
- long distance shot presenting centre of the field (L_C)
- long distance shot presenting right side of the field (L_R)
- long distance shot presenting left side of the field (L_L)
- long distance shot presenting spectators (L_S).

Taking into account the diversity of positions and orientations of the cameras around an arena and players on the pitch and the shots present in the broadcasted video, the indexing task is very complex and time consuming. For example, to detect a human in a close-up type of shot, different techniques may be used [7, 10]. All of them, however, deal only with fixed class of cases where, at least the human upper body is visible (i.e., not occluded or partially occluded upright human posture). This characteristic of the detector is not sufficient for the task where the human presented can wear different types of clothes (i.e., clothes which can in some ways affect the shape of human silhouette) like helmets or heavy body protectors as in ice hockey.

Scene class recognition algorithms based on SIFT [21] or HoG [10] visual features are performing quite well in this task but may be vulnerable to the different patterns of the background not presented during training. Moreover, these techniques require relatively a lot of time to calculate the descriptor which is always very long (especially for HoGs) and hard to interpret. This involves the usage of either SVMs that support very long input vectors or other techniques like bag of visual words or Fisher kernels [26]. Detection of other kind of shots could be even more difficult regarding the fact that the algorithm has to deal with different kinds of field sports. This is the reason why our procedures do not analyse very specific, local features and instead of this try to handle the problem with a fast global approach.

## The algorithm

The block diagram of the algorithm is presented in Fig. 2. The algorithm presented in the paper can be implemented in different variants. This section presents the procedure that acts as the basis for each variant. The feedback loop presented in the diagram acts differently depending on the type of the algorithm. Also, depending on the variant of the algorithm, the module responsible for collecting the partial descriptors performs different set of tasks—from acting like a simple buffer that pushes out all the data to partial descriptors gathering, averaging the results to one common representation.

**Fig. 3:**
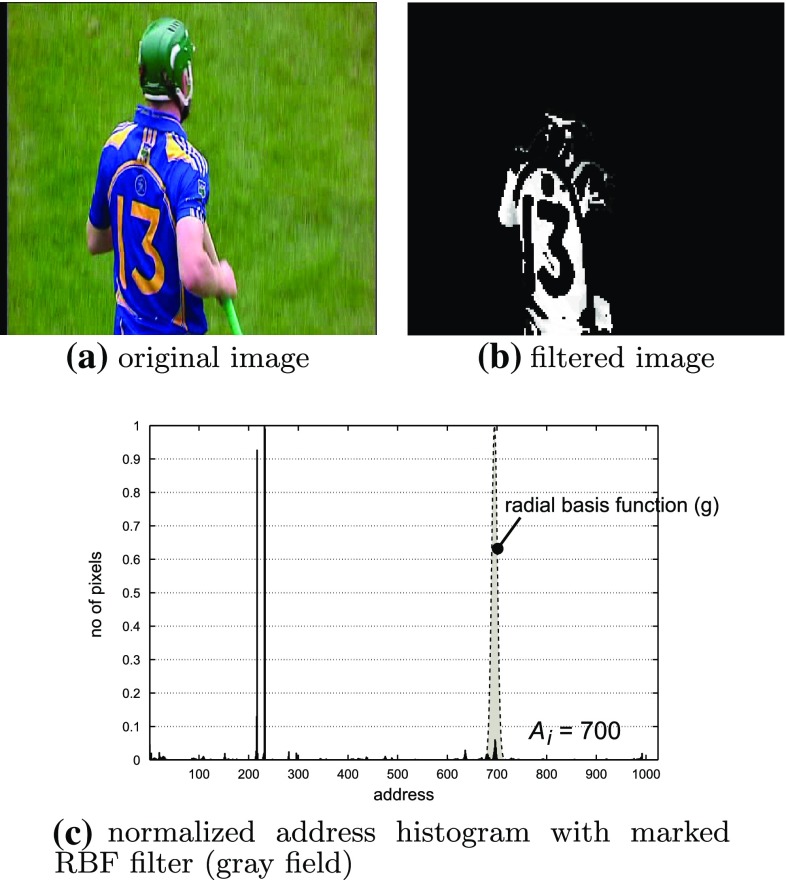
Visualization of the first two steps in scene detection algorithm

The underlying idea of the algorithm is to treat the image as it was a special kind of set of textures. Then, the very well-known Fourier transformation is used to describe the texture characteristics. The preprocessing steps are visualized in Fig. 3. Let $I$ be the input image shown in the Fig. 3a where colours are coded in HSV colour space. We convert each pixel to its address representation according to the following formula:

1 $$\begin{aligned} I_{x,y}^A=64H_{x,y}+16S_{x,y}+V_{x,y}+1 \end{aligned}$$

where $H_{x,y}, S_{x,y}$ and $V_{x,y}$ are quantized *H*, *S*, *V* coefficients to 16 (4 bits), 4 (2 bits) and 16 (4 bits) levels, respectively. Therefore, the resulting histogram has 1,024 bins (10 bits). Note that histograms calculated in this way (Fig. 3c) group similar colours with respect to their hue coefficient.

Now, let $g$ be a radial basis function (RBF) defined as follows:

2 $$\begin{aligned} g(A_i,x,y) = \exp \left[ -\frac{\left( I^A_{x,y}-A_i\right) ^2}{\sigma _{\text{ RBF }}^2}\right] \end{aligned}$$

where $A_i$ is the address at a given algorithm iteration (i.e., position of the $g$ function) and $\sigma _{RBF}^2$ is variance (span) of RBF. In results section, we analyse the influence of $\sigma _{RBF}^2$ to the accuracy of the description process. The meaning of $g(A_i)$ is that for a given address $A_i$ it processes address image $I_A$ (1) to detect colour-occurrence map presented in Fig. 3b. For further simplicity, the described process will therefore be described as:

3 $$\begin{aligned} I_{i}^{\text {RBF}}(x, y) = g(A_i, x, y) \end{aligned}$$

where $I^A$ is an address image with every pixel converted to its address representation accordingly to Eq. (1) and index $i$ denotes the position of $g(A_i)$ function (Fig. 3c).

To detect occurrence maps for following colour ranges, we create a loop in which the $g(A_i)$ function traverses full address histogram with step $A_s$, thus producing a set of grayscale images processed in further steps of the algorithm. Iteration process is also visible in the block diagram of the algorithm as a feedback loop (Fig. 2).

Colour-occurrence map (Fig. 3b) is then transformed with a 2-D Fourier transform $\mathcal {F}$ which gives us as a result the frequency representation of the particular colour distribution in the image (i.e., the colour layout is treated as it was representing a part of a texture in the image). The next step is filtering the Fourier representation with a set of Gabor filters [27]:

4 $$\begin{aligned} G_{K,L}\left( \omega , \theta \right) = \exp \left[ \frac{-\left( \omega -\omega _{K}\right) ^{2}}{2\sigma ^{2}_{\omega _{K}}} \right] \exp \left[ \frac{-\left( \theta -\theta _{L}\right) ^{2}}{2\sigma ^{2}_{\theta _{L}}} \right] \end{aligned}$$

where $K$ and $L$ are radial and angular indexes, respectively, $\omega _{K}, \theta _{L}$ are the polar coordinates of the filter centre and $\sigma ^{2}_{\theta _{L}}, \sigma ^{2}_{\omega _{K}}$ stand for width and height of the filter, respectively. Just like in [27], we are using a set of 30 functions that are geometrically similar and thus can be easily used to form banks of filters. Since we pay more attention to the low-frequency components of the Fourier transform, the sizes of the filters close to the origin of the Fourier space are significantly smaller than the ones whose centre is more distant.

The centres are spread across the space exponentially in radial and linearly in angular directions, respectively. Since the Fourier transform is symmetric with respect to the origin of its space, the bank of filters covers only the upper half of the space. The main property of this set of filters is that they have very good capabilities to capture local spatial frequencies of the image, thus uniquely represent it as a vector of features. Moreover, it has been proven, that Gabor feature space is robust to the most difficulties related to the image and video processing applications such as translation, scale and rotation (5):

5 $$\begin{aligned} G_{I'}\left( x_1,y_1;\,f,\theta \right) =G_{I}\left( ax_0, ay_0;\frac{f}{a},\theta -\phi \right) \end{aligned}$$

where $I$ and $I'$ denote original and transformed image, respectively, $x_1,y_1$ are new coordinates after translating image from location $x_0,y_0, a$ is a scaling factor, $\phi$ is a rotation angle and $f, \theta$ are the central frequency and the rotation angle of the Gaussian major axis and the plane wave, respectively, [16]. Thus, thanks to above properties Gabor filters have been used in range of applications dealing with problems like texture analysis or iris/face recognition.

The homogeneous texture descriptor vector proposed in [27] is composed of two parts: the logarithm of modules of Fourier coefficients and a logarithm of their standard deviation. Unlike this, we proposed a different representation of the information included in the Fourier domain.

In the resulting descriptor that we use the first coefficient is always a maximum over all iterations of $g$ function of logarithm all the moduli of Fourier coefficients in a single iteration:

6 $$\begin{aligned} D_E = \max _{i}\left( \frac{1}{N}\sum _{K,L}\log \left( 1+\left| {F}_i^{G_{K,L}}\right| \right) \right) \end{aligned}$$

Next, the mean value of the moduli of the Fourier coefficients located in the centre of Fourier space is taken:

7 $$\begin{aligned} D_C=\frac{1}{N}\sum _{i} \left| {F}_i^{\text {centre}}\right| \end{aligned}$$

where,

8 $$\begin{aligned} \left| {F}_{i}^{\text {centre}}\right| = \sum _{\omega =0}^{0.1}\sum _{\theta =0}^{\pi }\left| \mathcal {F} \left\{ I_{i}^{\text {RBF}}\right\} \right| \end{aligned}$$

The remaining part of the vector is split into two subsections: the first part is the mean of the Fourier coefficients under the given filter.

9 $$\begin{aligned} D_{\text {avg}}^{K,L} = \frac{1}{N}\sum _{i} \left| {F}_i^{G_{K,L}}\right| \end{aligned}$$

Since calculation of a standard deviation involves the knowledge about the mean value, it can slow down the descriptor extraction process significantly (two iterations over the set of values are needed). Thus, unlike in other algorithms based on the filtering with use of Gabor filters, we replace second part of descriptor which contains standard deviations of the mean values with a sum of logarithms of mean values.

10 $$\begin{aligned} D_{\text {log}}^{K,L} = \frac{1}{N}\sum _{i} \log \left( 1+\left| {F}_i^{G_{K,L}}\right| \right) \end{aligned}$$

where,

11 $$\begin{aligned} \left| F_i^{G_{K,L}}\right| = \sum _{\omega =0}^{1}\sum _{\theta =0}^{\pi }\left[ G_{K,L}|\omega | |\mathcal {F}\left\{ I_i^{\text {RBF}}\right\} |\right] \end{aligned}$$

and $|\omega |$ is a Jacobian between Cartesian and Polar frequency coordinates $|\omega |=\left| \sqrt{\omega _{x}^{2}+\omega _{y}^{2}}\right|$ and $N$ is a number of Gabor filters used.

The resulting descriptor is then represented by the following formula:

12 $$\begin{aligned} D = \left[ D_E, D_C, D_{\text {avg}}^{0,0} \ldots , D_{\text {avg}}^{4,5}, D_{\text {log}}^{0,0}, \ldots , D_{\text {log}}^{4,5} \right] \end{aligned}$$

### Real-time algorithm variant

Based on the linearity of the Fourier transform, we can merge all the results of single step calculations for the same value of $\sigma ^{2}_{\text {RBF}}$ into one result matrix by simply summing them and performing Gabor filtering only once at the end. So the resulting equation becomes ($M$ is the number of steps):

13 $$\begin{aligned} F=\frac{1}{M}\sum _{i=1}^{M}\mathcal {F}\left\{ I_i^{\text {RBF}}\right\} \end{aligned}$$

Thanks to performing the filtering step only once, we can achieve a very quick feature extraction method. In addition, calculations for different sizes of the $\sigma ^{2}_{\text {RBF}}$ factor can be done independently, thus we can combine the results to train and evaluate a set of SVMs for every given class. This technique allows us to choose the best performing combination of features and SVMs for every class. Results presented in this paper show that the proposed algorithm provides the same accuracy as sophisticated and slow feature extraction algorithms like SIFT [21] or HoG [10]. Note, that using an SVM as a classifier does not change the real-time character of the solution proposed herein. This is due to the fact that the time needed for SVM response given the calculated descriptors is negligible comparing to the descriptor extraction time (a single detection takes about 113 [$\upmu$s]).

However, some information about the scene detail is lost if we perform the feature extraction in the way described above. This is because we do not know if the given descriptor value was calculated based on multiple or one Fourier transform. Based on the experiments we performed, it can decrease the accuracy of the classifier for the images that contain high number of colourful details (e.g., shots that present spectators). On the other hand, sweeping the address histogram and performing Fourier transformation many times can be still quite expensive. For this reason, we also propose an alternative. The computational delay can be decreased by performing Fourier transformation and Gabor filtering only for the most important addresses (i.e., addresses where many pixels were allocated) and sort them in decreasing order based on this value. This way we can construct a fixed length descriptor with number of elements equal to $A\mathtt\,\,length (D)$, where $A$ is the number of the largest image areas.

### Bag of visual words algorithm variant

Given Eq. (3), we can set different values of $\sigma ^{2}_{\text {RBF}}$, without result concatenating techniques used in the real-time variant to create a set of feature vectors that will be further interpreted as a vocabulary of visual words (BoW). Moreover, this procedure can be used in a sub-window mode where for each sub-window of the image, the same procedure is calculated. Thus, depending on the number of different colours in the image, the resulting BoW can contain from several tens of words for images without high colour diversity, up to several thousands of words using multi-window approach on colourful images. To use an SVM with linear base function, we can transform the feature vectors with $\chi ^2$ kernel. This simplifies and speeds up the SVM training process. In addition, this approach does not need the post-train evaluation of the SVMs for different $\sigma ^{2}_{\text {RBF}}$ values since we have only one set of SVMs at the end of the training process.

In both cases, the execution times were significantly higher (5–20 times) than in the real-time variant of the algorithm, even when the extraction procedure is designed in a way, that it allows to split the computational burden into parallel threads.

### Classification

We took one of the most popular implementations of SVM train and predict procedures—the libSVM library [5]. It allows the same functionality in several coding languages such as C++, Java, MATLAB script etc. but since the time performance is crucial issue in our case we tested its C++ implementation. As for the training, it also allows to create a whole range of SVM kernels: linear, polynomial, radial, sigmoid and types: C-SVC, nu-SVC, epsilon-SVR, nu-SVR with appropriate parameters for each type. To determine the optimal SVM configuration, we performed in-loop training of SVM for each given class. All of the inferred optimal SVMs were C-SVC SVMs with RBF kernel.

**Fig. 4:**
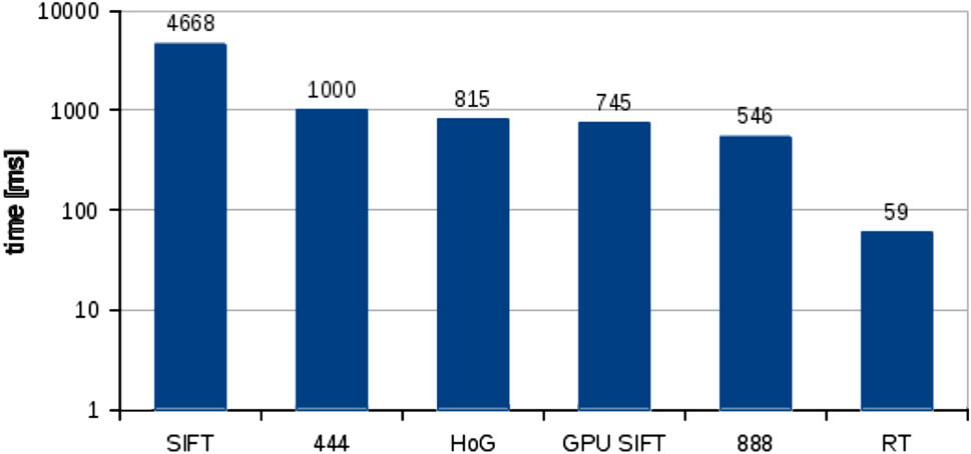
Comparison of calculation times of the algorithm presented in the paper with one of the most commonly used algorithms nowadays

## Results

The results presented herein are split into three parts to improve the readability of the evaluation. First, we focus on the accuracy of the system where the comparison of the accuracy, recall and precision of the algorithm as well as for some of the algorithms that comprise state-of-the art is presented. Then, the performance of the algorithm is analysed. Also, the comparison between available algorithms and our implementation, plus some investigation on the influence of the algorithm’s parameters on the calculation time is presented. Finally, we present some investigation of the accuracy of the algorithm for images of different resolution so that the robustness of the procedure is evaluated.

### Dataset

For the purposes of developing our approach, an image dataset was created consisting of a variety of field sport genres including rugby, ice hockey, soccer and Gaelic football, hurling, cricket and basketball. To ensure generality, the content was captured from various broadcast sources at different resolutions [13]. In all, 30 h of content was captured. This was split into two subcorpuses. The training corpus was to be used in developing model hypotheses, and the testing corpus purely for evaluation purposes. Both were manually annotated, such that advanced knowledge of all event locations was ascertained and subsequently used as our ground truth.

**Fig. 5:**
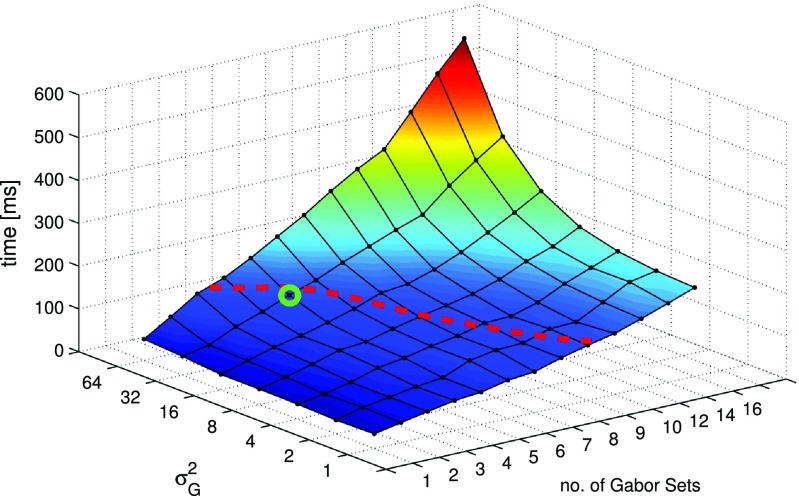
The impact of the number of sets of Gabor filters and the variance of sweeping RBF on the performance of the algorithm; *red*, *dashed line* marks the real-time region; the marked point denotes parameters that provide the highest accuracy

### Accuracy

Table 1 shows a general comparison between the state-of-the art algorithms and the set of algorithms presented in the previous section. ACC. REC. and PRE. denote accuracy, recall and precision, respectively. The first column shows the classes we introduced in Sect. 2. The next two columns present the results for state-of-the art algorithms. First, we took a very well-known and widely used SIFT algorithm as a representative of general image/object recognition approach. Since the type of broadcast video we are dealing with consists of shots that present humans, HoG was chosen as a representative of one of the best algorithms for human detection [10]. For both experiments, we used well-known libraries [34] to implement feature extraction module together with bag of words technique based on 1,000 clusters. The following six columns show the results of the proposed algorithm with different combinations of sweeping radial basis function, followed by the column that gathers the best results for every row. The rationale behind this, is that classifiers that vary with the size of the input data can be used for different classes once they have been trained and evaluated. The three numbers that denote a particular set-up of a sweeping RBF can be read as follows: start variance, variance step and final value of variance. $s$ denotes that the descriptor values were scaled before the SVM training process. For example, 228 s means that the start variance was 2, the algorithm performed Fourier transform 512 times—1,024/2, then it changed variance by 2 (variance step) and repeated the same transformation, this time 256 times—1,024/4. These steps were repeated until the variance reached its final value—8. In this particular example, before the SVM training process the feature vector scaling algorithm was invoked. The next two columns show the result for the bag of words algorithm variant. The *_MW* extension denotes “multiple window” version of this variant. Finally, the last column denoted as RT shows the results for the best performing real-time variant of our algorithm.

**Table 1:** The general comparison of the tested algorithms

	*HoG*	*SIFT*	*228*	*228s*	*444*	*444s*	*888*	*888s*	*BEST*	*BoVW*	*BoVW_MW*	RT
CL_H	88.2	88.5	**87.7**	84.2	87.1	87.1	84.9	84.2	87.7	83.5	83.4	84.6
CL_H_S	90.7	89.8	90.9	90.7	90.8	**93.6**	90.5	87.7	93.6	86.4	87.6	85.9
CL_H_C	90	90.7	90.1	90	88.6	**90.8**	88.9	88.8	90.8	89.9	86.5	86.4
CL_WU	83.6	81.4	**85.8**	84.9	84.5	85.7	82.6	83.2	85.8	85.6	83.9	82.6
CL_WU_S	84.4	81.1	77.6	78.7	74.8	**81**	77.2	75.5	81	82.1	75.5	77.6
CL_WU_C	83.1	85.5	85.4	85.1	**85.5**	84.9	82.4	83.5	85.5	89.1	84.7	85.6
S_P	79.6	86.1	77.1	74.9	78.2	**81.7**	74.6	74.1	81.7	78.5	74.5	78.1
S_P_S	84.4	88	88.8	89.4	89.7	**91.1**	87.6	88.3	91.1	88.1	86.3	90.7
S_P_C	83.8	85.1	83.8	81.8	79.9	**84**	79.3	73.9	84	85	81.4	78.6
S_S	81.4	87.7	83.4	82.6	82	**85.1**	80.7	76.4	85.1	79	81.6	82.7
L_C	90.6	92.1	89	84.9	87.2	**90.9**	87.8	88	90.9	90.4	90.4	91.7
L_L	94.3	92.5	89.1	79.1	88.9	82.4	**89.1**	89.4	89.1	82.4	88.9	81.3
L_R	97.7	91.3	80.9	67.3	**84.9**	79	81.3	76	84.9	65.2	81.6	78.1
L_S	94	92.6	94.4	94.1	**95.9**	95.5	95.1	93.7	95.9	93.2	93.9	94.1
**ACC.**	**87.6**	**88**	86	83.4	85.6	86.6	84.4	83	**87.7**	84.2	84.3	84.1
**REC.**	**87.2**	**88.3**	87	81.2	87.4	85.6	86.3	87.8	**90.9**	85.5	85.8	84.2
**PRE.**	**88.5**	**87**	84.9	85.2	84.1	86.4	83.1	80.6	**89.3**	82.9	83.1	82.5

Bold values stand for the best results in a row between version 228 and 888s of the algorithm. They are also collected in BEST column

Note, that all the variants of the algorithm proposed herein perform at a very similar level of accuracy. The real-time variant, since more constrained for performance reasons, is about three percent less, but the result is high enough to make it suitable for fast shot/scene recognition.

The two last rows show the average recall and precision for all classes. Note that for conciseness particular values for the classes are not shown. The best option of our algorithm (the one that comprises of different extraction algorithms for different classes) outperforms the state-of-the art algorithms. Based on this, we can make an interpretation, that the relevance of the algorithm proposed herein is better than existing, state-of-the art algorithms.

### Time performance

Clearly, the most complex module in the system is the one that implements the Fourier Transform. The algorithm used in our application is the one that involves Fast Fourier Transformation (FFT) for this purpose ($O(N \log (N))$). The next most complex module is the one that calculates Gabor filtering based on convolution with pre-calculated filters ($O(N_1 \log (N_1))$). Complexities of $I^A$ and *Descriptor collector* blocks are linear due to the fact that address representation of an image is calculated based on look-up table (LUT) and *Descriptor collector* block just sums and stores results from Gabor filtering. Since $N >> N_1$, we can say that the complexity of the system is dominated by FFT module.

Figure 4 shows the calculation time result comparison of the scene recognition algorithms used in our experiments. All the measured times are the application times without the time spent in the operating system kernel. Since the measurements are slightly affected with small inaccuracy (about 2 %), the results presented are the mean values of a hundred runs of a particular algorithm. The more detailed breakdown of the influence of the algorithm’s parameters on the execution time is presented later. The time shown in Fig. 4 for the real-time variant is for the accuracy presented in the last column in the Table 1. Since HoGs were calculated for every pixel in the image the resulting vector is very long, even for images of reduced resolution (4,356 elements for $100\times 100$ pixel image). Even then the execution time was relatively long in comparison to the algorithm we are proposing for the same task.

As can be seen, the proposed algorithm significantly outperforms the existing solutions. Even the longer, more accurate versions are much quicker than SIFT. The time for SIFT compiled with CUDA support [32] is also much slower than the real-time version of the algorithm. In sports video broadcasts, a single shot is usually presented for at least a few seconds [35], so it is necessary only to process every other frame. The real-time version is capable of processing video at up to seventeen frames per second, and is therefore suitable for real-time applications on sports video broadcasts.

Figure 5 shows the impact of the number of Gabor sets and the variance of the sweeping RBF on the performance of the procedure. Again, only the time of the application execution was measured. Taking into account, that the particular shot presented to the user lasts for at least a few seconds [35], for better accuracy and robustness of the algorithm, several frames per second were analysed. This requires, that the algorithm can fulfill its task in less than about 100 ms. For this reason, we can introduce a boundary (red, dashed line) in Fig. 5 that separates the real-time region of the parameters of the algorithm.

**Fig. 6:**
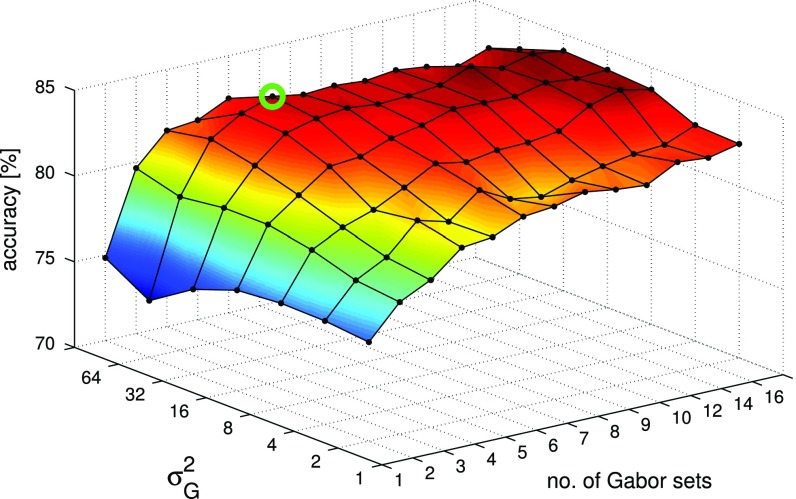
The impact of the number of sets of Gabor filters and the variance of sweeping RBF on the accuracy of the algorithm; the marked point denotes parameters that provide the highest accuracy

Figure 6 shows how the accuracy of the procedure depends on the two parameters presented earlier. In general, we can say that the higher the amount of the Gabor sets, the higher the accuracy. For the variance of the RBF, the algorithm provides the best performance when it is equal to 32. Taking into account the consideration about the time performance, we can easily choose the set of parameters that fulfill both: the real-time and high accuracy requirements. This point is marked in Figs. 5 and 6 as a green circle. The performance of the algorithm with this set of parameters is presented in Table 1 as the RT algorithm option.

We have also tested a response time of the SVM classifiers. The time performance of the predict algorithm is so efficient that, in comparison to the extraction time of a descriptor, can be neglected (113 [$\upmu$s]). Concluding, even if there are multiple SVMs in the system, the overall time overhead will not hamper real-time behaviour.

### Robustness

**Fig. 7:**
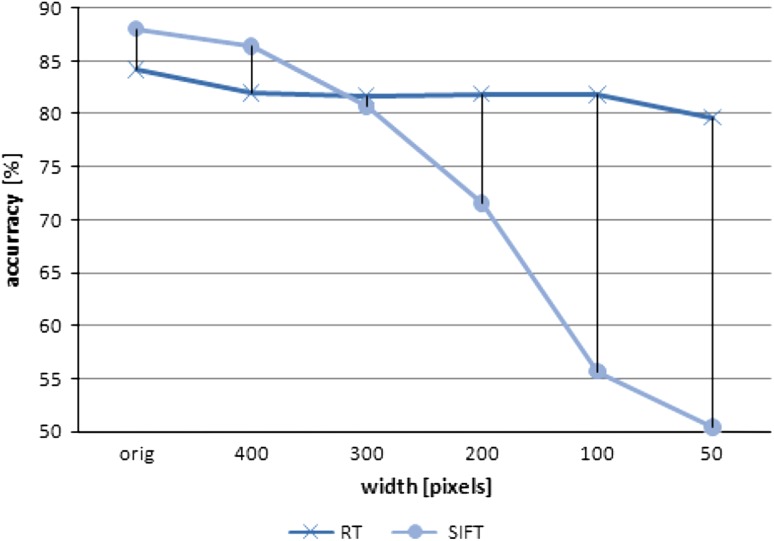
The impact of the size of the image (width) on the accuracy of the algorithm

One of the most important features of the scene recognition algorithms proposed by different researchers is robustness to changes in resolution. This is true especially for the field sports videos analysis case since we cannot expect that all the videos will be of the same frame size (particularly in the case of internet videos). It is very unlikely that processed video would be transformed with any kind of affine transformations since the human visual system is very sensitive to the change of the aspect ratio. For this reason, only the robustness to the resolution of the video was investigated. Figure 7 shows the results of this experiment. Only one variant of the algorithm was chosen (RT), since all variants of the algorithm presented herein are based on the same preprocessing step, they feature the same robustness characteristics. As it can be seen, the SIFT transform is much less accurate for videos of low resolution, considering the fact that in the case when the width of the video reaches about 200 pixels the accuracy of the classifier becomes close to random. This is highly undesirable situation since very often Internet videos are of much poorer than HD quality. The RT variant does not lose its recognition ability so quickly and, even for small videos, retains almost unchanged accuracy.

### Memory requirements

As real-time applications usually run under constrained system resources, it is desirable to analyse the memory footprint of this kind of algorithms. Figure 2 shows several stages of the algorithm where implementation of the buffers containing input image or intermediate results of the algorithm is needed. Note, that the result of the algorithm itself very little depends on the resolution of the input image (Fig. 7). However, to reduce memory, footprint images above $512\times 512$ elements are resized to this resolution so that the input buffer has $512\times 512 \times 3 =$ 786,432 bytes. This is save tradeoff between the number of details in the image and the requirement for memory. The next block where $I^A$ is stored takes $512\times 512 \times 1.25 =$ 327,680 bytes (note, that each cell of the $I^A$ image takes 10 bits). Following block stores temporary result of colour-occupancy map $I^{\text {RBF}}_i$ as a grey-scale image ($262,144$ bytes). As $I^{\text {RBF}}_i$ is needed only for FFT calculation the “in place” FFT and Gabor filtering variants can be used that do not require additional memory. The width of the *Descriptor collector* buffer depends on the variant of the algorithm and takes the range from 150 to 800 bytes.

## Conclusion

The paper presents a novel approach to recognize a scene presented in the image. We have proposed different variants of the algorithm ranging from bag of visual words to simple real-time implementation which takes only the most important areas of similar colour into account. All the variants provide similar accuracy which is comparable to very well-known image indexing techniques like SIFT or HoGs. The algorithm is very suitable for the scene recognition task thanks to its speed (complexity is equal to $O(N \log (N))$) and also robustness for variable image resolution, thus, making it a good candidate for real-time video indexing systems. The usage of the Fourier transform is an advantage in terms of hardware implementations of video processing systems since there exist a number of highly optimized hardware IP cores that implement it [23, 24]. Moreover, these kinds of modules are already used in a range of signal processors.

**Fig. 8:**
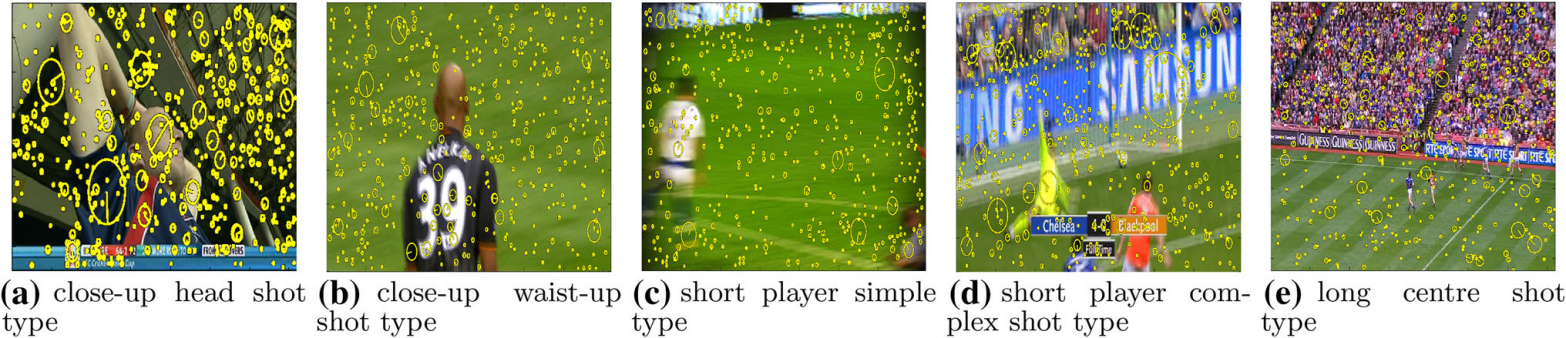
Exemplary SIFT misclassifications in particular classes; SIFT features are marked with *yellow circles* (captions denote the correct class)

**Fig. 9:**
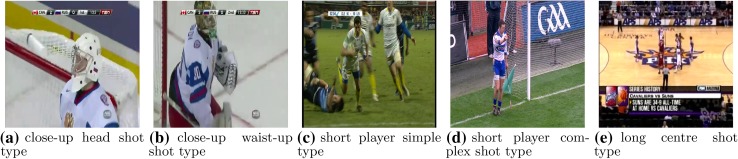
Exemplary misclassifications of the algorithm presented in the paper in particular classes (captions denote the correct class)

**Fig. 10:**
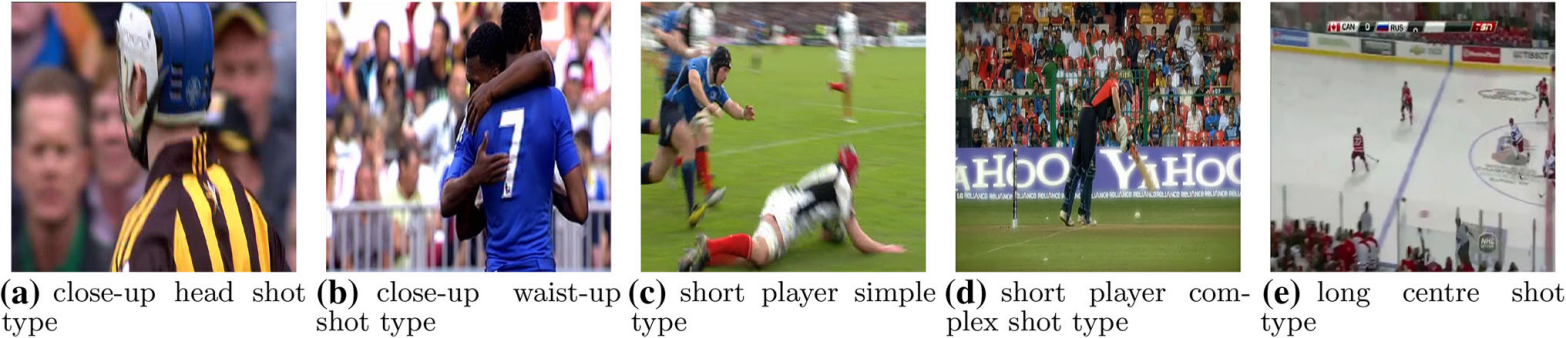
Exemplary correct classifications of the algorithm presented in the paper in particular classes (captions denote the correct class)

In contrast to SIFT and HoG, a global approach is taken during extraction of the feature vector. The image is not processed locally to detect key points. Instead, it is treated as a set of related objects, that can be characterized based on their colour, that collectively represent uniqueness of the scene. In other words, the approach taken, is similar to the one used in the task of texture recognition. This makes it quick (image is not processed locally) and robust to a variety of image transformations (translation, rotation, scaling). Global approach also makes the algorithm different in the way it mis-classifies the images. Figures 8 and 9 show the differences in misclassifications between SIFT and our approach. Since SIFT describes objects locally, it is vulnerable to the specific background that consists of a number of points recognized as key points by the transform (corners). Figures 8a, d, e show examples of these kinds of scenes. In the first case (Fig. 8a), a person presented is on the background of the ceiling of the arena that consists of the cross-type construction. In the subsequent two cases (Fig. 8d, e), local features are found in the spectator’s area and in the side bounds of the pitch. In all cases, the number of the non-significant key points exceeds the number of key points detected in the objects that were supposed to be recognized. Two remaining images (Fig. 8b, c) present a common situation where after a score the camera focuses on the player. These kinds of shots are often quite dynamic, thus, usually the boundary of the player’s silhouette presented is not clearly visible. That makes key point detection difficult, since it is based on Harris corner detector. However, our algorithm can deal with this. Figure 9a, b presents situations where player wears a jersey which is of very similar colour than the ice rink. In addition, in the first case, a helmet colour is also the same which makes the shot presented extremely hard to recognize. In Fig. 9c, a number of players are present close to each other, thus decreasing the ability to distinguish them separately from the background. Figure 9d, e presents shots that contain too many details compared to the object to be detected, to be recognized correctly.

Figure 10 shows exemplary correct classifications of the algorithm proposed in the paper. As can be seen, it can deal with a variety of effects that make correct scene recognition a complex task (e.g., partial/global occlusions, perspective, complex background, etc.). The algorithm is highly useful in the cases where we cannot expect presented objects (in our case players) to be in the certain position or take certain pose or shape. Figure 10a–d show these kinds of situations. In these cases, existing state-of-the art algorithms like Viola-Jones head detector or HoG human detector simply cannot work because of the facts that the player may wear a helmet, could be presented from the back or have non-upright posture. Also the fact that our algorithm focuses not on colour, but on the layout of it or its texture means that it is suitable for field sports that are not only played on the grass but also on other fields like ice or parquet, for example Fig. 10e.

It has been demonstrated that the algorithm presented herein can be successfully used in various scene recognition applications and can be implemented in a number of varieties. The paper presents three of the most interesting, in our opinion, variants: standard algorithm, bag of visual words variant and real-time variant. Each of them results in different time performance and efficiency but they all share the same preprocessing stem (Fourier transform plus sweeping through the specific colour histogram with a Gaussian function). From our point of view, the most interesting ones are the standard option and the real-time one. Since the time requirement is very often a limitation in various systems, we proposed a quick and deterministic (in response time) version of the algorithm. It is a little less accurate than the standard variant, but the calculation time significantly outperforms any other algorithm we compared in this paper. For repeatability of the experiment, we have put our database online so that it can be accessed by anyone interested in this task. According to our knowledge, it is the first case where a database for this task is available.

The fact that the response time is highly deterministic, makes this algorithm suitable for hard real-time systems. Moreover, since only colour is analysed instead of edges, we do not have to rely on the quality of the frame presented. This is highly desirable in video analysis where scenes are dynamic. All the above features make the algorithm suitable in a range of applications where our choice was the shot-type detection of field sports videos. Thus, thanks to implementation of this algorithm applications that are focused on tasks such as event detection, highlight extraction, video skimming, and table of content extraction can work faster and more accurately.

## Future work

The work presented herein is a part of a Visual Content Recognition System (VISION). Since it has been shown [29] that correctly recognized scenes in broadcast video can lead to efficient event recognition, we plan to integrate our algorithm into the video browsing engine developed especially for this project. We also plan to use other features like the score presented in the scoreboard, and audio energy, to improve the performance and robustness of the event extraction engine.
